# EGF-activated PI3K/Akt signalling coordinates leucine uptake by regulating LAT3 expression in prostate cancer

**DOI:** 10.1186/s12964-019-0400-0

**Published:** 2019-07-25

**Authors:** Blake K. Zhang, Anne M. Moran, Charles G. Bailey, John E. J. Rasko, Jeff Holst, Qian Wang

**Affiliations:** 1 Centenary Institute, University of Sydney, Camperdown, Australia; 2 Sydney Medical School, University of Sydney, Camperdown, Australia; 3 Gene & Stem Cell Therapy Program Centenary Institute, University of Sydney, Camperdown, Australia; 4 Cell and Molecular Therapies, Royal Prince Alfred Hospital, Camperdown, Australia; 5 Translational Cancer Metabolism Laboratory, Lowy Cancer Research Centre, School of Medical Sciences and Prince of Wales Clinical School, University of New South Wales, Sydney, Australia; 6 Origins of Cancer Program Centenary Institute, University of Sydney, Camperdown, Australia

**Keywords:** EGF, PI3K/Akt signalling pathway, L-type amino acids transporter 3, LAT3, SLC43A1, Prostate cancer

## Abstract

**Background:**

Growth factors, such as EGF, activate the PI3K/Akt/mTORC1 signalling pathway, which regulates a distinct program of protein synthesis leading to cell growth. This pathway relies on mTORC1 sensing sufficient levels of intracellular amino acids, such as leucine, which are required for mTORC1 activation. However, it is currently unknown whether there is a direct link between these external growth signals and intracellular amino acid levels. In primary prostate cancer cells, intracellular leucine levels are regulated by L-type amino acid transporter 3 (LAT3/SLC43A1), and we therefore investigated whether LAT3 is regulated by growth factor signalling.

**Methods:**

To investigate how PI3K/Akt signalling regulates leucine transport, prostate cancer cells were treated with different PI3K/Akt inhibitors, or stable knock down of LAT3 by shRNA, followed by analysis of leucine uptake, western blotting, immunofluorescent staining and proximity ligation assay.

**Results:**

Inhibition of PI3K/Akt signalling significantly reduced leucine transport in LNCaP and PC-3 human prostate cancer cell lines, while growth factor addition significantly increased leucine uptake. These effects appeared to be mediated by LAT3 transport, as LAT3 knockdown blocked leucine uptake, and was not rescued by growth factor activation or further inhibited by signalling pathway inhibition. We further demonstrated that EGF significantly increased LAT3 protein levels when Akt was phosphorylated, and that Akt and LAT3 co-localised on the plasma membrane in EGF-activated LNCaP cells. These effects were likely due to stabilisation of LAT3 protein levels on the plasma membrane, with EGF treatment preventing ubiquitin-mediated LAT3 degradation.

**Conclusion:**

Growth factor-activated PI3K/Akt signalling pathway regulates leucine transport through LAT3 in prostate cancer cell lines. These data support a direct link between growth factor and amino acid uptake, providing a mechanism by which the cells rapidly coordinate amino acid uptake for cell growth.

**Electronic supplementary material:**

The online version of this article (10.1186/s12964-019-0400-0) contains supplementary material, which is available to authorized users.

## Background

Binding of growth factors to the extracellular ligand binding domain of their membrane-bound receptors leads to a conformational change of the receptors, thereby activating tyrosine or serine/threonine kinase domains. This activation enables the recruitment of diverse substrates, propagating signals that mediate a plethora of cellular activities ultimately leading to cell growth [1]. The uptake and metabolism of extracellular nutrients is one of the most critical cellular activities required to provide the building blocks and energy necessary to produce new cells [2]. While numerous studies have suggested that growth factors can regulate uptake of nutrients, whether by transporters, or by macropinocytosis, a direct link to transport has not yet been established [3–5].

Growth factors and their receptors are commonly increased in a variety of cancers, with expression of epidermal growth factor (EGF) and its receptor (EGFR) significantly increased in prostate cancer [6]. Binding of EGF to EGFR stimulates downstream signalling pathways including the mitogen-activated protein kinase (MAPK) and phosphoinositide 3 kinase (PI3K)/Akt pathways. In addition, the PI3K/Akt signalling pathway is commonly activated in cancers, either through activating mutations or inactivation of the tumour suppressor phosphatase and tensin homolog (PTEN) [7, 8]. In prostate cancer, up to 70% patients have PTEN mutation or deletion [9], thereby allowing unconstrained growth factor activated PI3K/Akt signalling, cell proliferation and tumour growth.

The PI3K/Akt signalling axis activates mechanistic target of rapamycin complex 1 (mTORC1) through phosphorylation, thus negatively regulating tuberous sclerosis complex 1/2 (TSC1/2) formation and releasing Rheb, a GTPase activating protein (GAP), to bind to the kinase domain of mTORC1 on the surface of lysosomes, leading to mTORC1 activation [10]. In addition, intracellular levels of free amino acids, in particular leucine, arginine and glutamine, regulate mTORC1 activation [11, 12]. Amino acids sufficiency can be sensed by mTORC1 through the interaction between Rag GTPase heterodimers and Ragulator on the surface of lysosomes [13–17]. Sestrin2 has been identified as a leucine sensor upstream of mTORC1 by binding with leucine which is required for mTORC1 activation [18, 19]. Thus, mTORC1 integrates upstream signalling pathways as well as amino acid availability to mediate protein synthesis, cell growth and proliferation.

The supply of intracellular amino acids is mediated by amino acid transporters, which are commonly upregulated in cancer cells. One such transporter, LAT3, mediates uptake of large neutral branched chain amino acids (BCAA) including leucine. LAT3, encoded by the gene *SLC43A1*, was originally named prostate cancer overexpressed gene 1 (POV1) [20], and was later identified as a major uniporter of leucine, isoleucine, valine, phenylalanine and methionine [21]. Human LAT3 is predicted to contain 12 transmembrane (TM) domains. A long intracellular loop between transmembrane domains 6 and 7 contains putative serine phosphorylation sites and a tyrosine phosphorylation site [22]. LAT3 protein expression is high in primary and recurrent prostate cancer, driven by direct androgen receptor (AR) transcription [23, 24]. Increased LAT3 levels result in increased intracellular leucine levels and subsequent activation of mTORC1 [23, 24]. Moreover, shRNA knockdown of LAT3 blocks leucine uptake and cell growth in prostate cancer cell lines both in vitro [23] and in vivo [24].

Since both the growth factor-activated PI3K/Akt signalling pathway and amino acid transporters are required for mTORC1 activation, we investigated whether there were any direct links between PI3K/Akt signalling and LAT3 activity. In this study, we show that EGF-activated PI3K/Akt signalling directly regulates leucine transport in prostate cancer cells by stabilising LAT3 expression on the cell surface. These data suggest that there is a coupling of growth signals to amino acid uptake in prostate cancer, which may provide new avenues to control cancer cell growth, and should be examined across a range of transporters and cancer types.

## Materials and methods

### Cell lines

Human prostate cancer cell lines LNCaP-FGC and PC-3 were purchased from ATCC (Manassas, VA, USA). We used low passage original stocks, and confirmed LNCaP and PC-3 cell identity by short tandem repeat profiling in 2014 (Cellbank, Australia). Cells are tested monthly by PCR to ensure they are free of mycoplasma contamination. Cells were cultured in RPMI 1640 medium (Invitrogen) containing 10% (v/v) fetal bovine serum (FBS), penicillin-streptomycin solution (Sigma-Aldrich) and 1 mM sodium pyruvate (Invitrogen). Cells were maintained at 37 °C in a fully humidified atmosphere containing 5% CO_2_.

### Leucine uptake assay

The [^3^H]-L-leucine uptake was performed as detailed previously [23]. Briefly, cells were cultured in 6-well plates in RPMI media. After serum starvation for 2 h, cells (3 × 10^4^/well) were incubated with 0.3 μCi [^3^H]-L-leucine (200 nM; PerkinElmer) in leucine-free RPMI media (Invitrogen) in the absence or presence of EGF (10 ng/mL, Sapphire Bioscience) or dialyzed FBS, or inhibitors, including MK2206 (10 μM, Selleck Chemicals), LY294002 (20 μM, Merck), 2-amino-bicyclo [2,2,1] hepta-2-carboxylic acid (BCH, 10 mM, Sigma), rapamycin (50 nM, Merck), JPH203 (10 μM, Selleck Chemicals), for the required time at 37 °C. Cells were collected, transferred to filter paper using a MicroBeta FilterMat-96 Cell Harvester (PerkinElmer), dried, exposed to scintillation fluid and counts were measured using a MicroBeta^2^ liquid scintillation counter (PerkinElmer).

### Western blot

Cells were seeded at a density of 5 × 10^5^ in 6-well plates, allowed to adhere overnight, before incubation with EGF and/or inhibitors, MK2206, LY294002, BCH, rapamycin, MG132 (50 μM, Sigma) for required time. Cells were lysed by addition of lysis buffer (200 μL) with EDTA-free Protease Inhibitor Cocktail III (cOmplete™; Roche Diagnostics) and Phosphatase Inhibitor Cocktail (100×, Cell Signalling Technology). Equal protein (micro-BCA method; Pierce™, Thermo Scientific) was loaded on 4–12% gradient SDS-PAGE gels (Invitrogen), electrophoresed and transferred to PVDF membrane (Millipore). The membrane was blocked with 2.5% (w/v) bovine serum albumin (BSA, Sigma-Aldrich) in PBS containing 0.1% (v/v) Tween 20 (0.1% PBS-T), and incubated with the primary antibodies overnight. The next day, membrane was washed with 0.1% PBS-T three times and incubated with secondary antibodies. After washing, the membrane was visualised using enhanced chemiluminescence reagents (ECL, Pierce™, Thermo Scientific) on a ChemiDoc™ TOUCH imager (Bio-Rad). Antibodies used in this study include phospho-P70S6K (Thr398, #9205, 1:1000), P70S6K (#9202, 1:1000), phospho-Akt (Ser473, #4051, 1:1000), Akt (#9272, 1:1000), Na, K-ATPase (#3010, 1:1000) from Cell Signalling Technology (CST); ubiquitin (ab7780, 1:1000), glyceraldehyde-3-phosphate dehydrogenase (GAPDH, ab8245, 1:2000) from Abcam. LAT3 rabbit polyclonal antibody was generated against a peptide (TGGKERETNEQRQ) from mouse LAT3 (The Institute of Medical and Veterinary Science, Adelaide, Australia) and affinity purified from rabbit serum [22]. Horseradish peroxidase (HRP)-conjugated donkey anti-mouse IgG (AP192P, 1:5000), and donkey anti-rabbit IgG (AP182P, 1:5000) from Millipore were used as secondary antibodies. Densitometry of western blots was analysed by ImageJ (NIH).

### Immunofluorescent staining

Cells were seeded on BD Falcon™ culture slides (Becton Dickinson) at a density of 1 × 10^4^ cells/well and allowed to adhere overnight. Fresh media was added, and the cells were incubated for 24 h before fixation using 4% (w/v) paraformaldehyde (Sigma-Aldrich) for 20 min, and permeabilisation using 0.1% PBS-T for 15 min. Cells were washed and incubated with 5% (v/v) normal goat serum in 2% (w/v) BSA/PBS for 30 min before addition of the LAT3 antibody at 4 °C for overnight incubation. The cells were washed in PBS before addition of a goat anti-rabbit IgG conjugated with AlexaFluor 594 (Invitrogen, A12381) for LAT3 antibody or a goat anti-mouse IgG conjugated with AlexaFluor 488 (Invitrogen, A12379) for pAkt antibody for 1 h at room temperature, and nuclear staining with DAPI (5 μg/mL, Thermo Fisher) for 5 min. Cells were washed in PBS, and coverslips were immersed in glycerol and placed on a slide, and visualized using a DeltaVision confocal microscope (GE Healthcare Life Science).

### Proximity ligation assay (PLA)

Cells were seeded on coverslip which has been immersed in poly-L-lysine (Sigma-Aldrich) at a density of 5 × 10^5^ cells/well and allowed to adhere overnight. Cells were incubated in serum free media for 30 min, followed by EGF (10 ng/μL) treatment for 30 min. The cells were fixed using 4% (w/v) paraformaldehyde for 20 min, and permeabilisation using 0.1% PBS-T for 15 min. Cells were washed and incubated with 5% (v/v) normal goat serum in 5% (w/v) BSA/PBS for 30 min before addition of the rabbit LAT3 antibody (1:50) and mouse pAkt antibody (1:50) at 4 °C overnight. For negative control, mouse pAkt antibody and rabbit IgG (Santa Cruz, sc-2027) or rabbit LAT3 antibody and mouse IgG (Santa Cruz, sc-2025) were added (1:50). As a positive control, pAkt and Akt antibody (1:50) were added. PLA was performed using the DUOlink™ kit (OLINK, Uppsala, Sweden) following the manufacturer’s instruction. The cells were visualized using a DM6000B microscope (Leica, Germany). Quantification of PLA signals was measured using Volocity software (Version 6.3, PerkinElmer).

### Cell surface protein isolation

LNCaP cells were cultured in T75 flasks with RPMI 1640 medium containing 10% FBS, penicillin-streptomycin solution and 1 mM sodium pyruvate to reach 90–95% confluence. LNCaP cells were incubated with serum free media for 2 h, followed by EGF (10 ng/μL) treatment for 30 min. Surface protein isolation was performed using the Pierce™ Cell Surface Protein Isolation Kit (Thermo Scientific) following the manufacturer’s instructions. Briefly, cells were biotin-labelled, harvested, and lysed, and NeutrAvidin resin was used to isolate the protein. Protein was eluted with SDS-PAGE sample buffer (NuPAGE™ Life Technologies) containing 50 mM 1,4-dithiothreitol (DTT, Sigma-Aldrich). The eluate was analysed by SDS-PAGE and western blot.

### Immunoprecipitation

LNCaP cells were seeded at a density of 5 × 10^5^ in 6-well plates and allowed to adhere overnight. The next day, cells were pre-treated with MK2206 or MG132 (50 μM, Merck) in serum free RPMI 1640 media for 30 min, before addition of EGF (10 ng/μL) to each well for 30 min. Cells were lysed by addition of lysis buffer with Protease Inhibitor Cocktail and Phosphatase Inhibitor Cocktail. Protein concentration of cell lysates was determined by the micro-BCA method. Cell lysates were incubated with magnetic protein A/G beads (Surebeads™ Bio-Rad) coupled with LAT3 antibody or rabbit IgG at 4 °C overnight. Protein-bound beads were washed with E1A buffer (50 mM HEPES, 150 mM NaCl, 0.1% NP-40, 1 mM Na_3_VO_4_) three times. Protein was eluted with sample buffer (NuPAGE™, Life Technologies) containing 0.1 M DTT, heated at 85 °C for 5 min, before SDS-PAGE and western blot. After examining ubiquitin levels, the blot was stripped using re-blot plus strong antibody stripping solution (Millipore). Then, the blot was incubated in blocking buffer (2.5% BSA in PBS-T) for 30 min and re-blotted with LAT3 antibody overnight. The predominant LAT3 present in the immunoprecipitate was ubiquitinated LAT3 (~ 90 kDa), similar to a previous report for other transporters [25], which may be due to altered conformation of the antibody binding sites on the antigen due to detergents in the immunoprecipitation buffer [26].

### Knockdown of LAT3

LAT3 shRNA knockdown was performed as previously described [23]. Briefly, empty vector pLKO.1 or containing short hairpin (shRNA) targeting LAT3 (CCGGCAAATCCATCAGACCACGCTACTCGAGTAGCGTGGTCTGATGGATTTGTTTTTG) was mixed with pMDLg/prre, pRSVRev and pMD2.VSV-G packaging plasmids and the calcium phosphate precipitation method used to transfect 70% confluent HEK293T cells [23]. After 8 h, the media was changed, and viral supernatant was collected and filtered 24 h later. The viral supernatant was used to transduce 70% confluent LNCaP with 8 μg/mL polybrene. After 2 days, transduced cells were selected by 10 μg/mL puromycin for 7 days. Expression of the LAT3 protein was determined using SDS-PAGE and western blot as described above.

### Statistical analysis

Data are shown as mean ± SEM. All experiments were performed with at least 3 biological replicates. All experiments were analysed by two-tailed student’s t-test or two-way ANOVA or one-way ANOVA in GraphPad Prism 6.

## Results

### PI3K/Akt signalling pathway regulates leucine transport

**Fig. 1:**
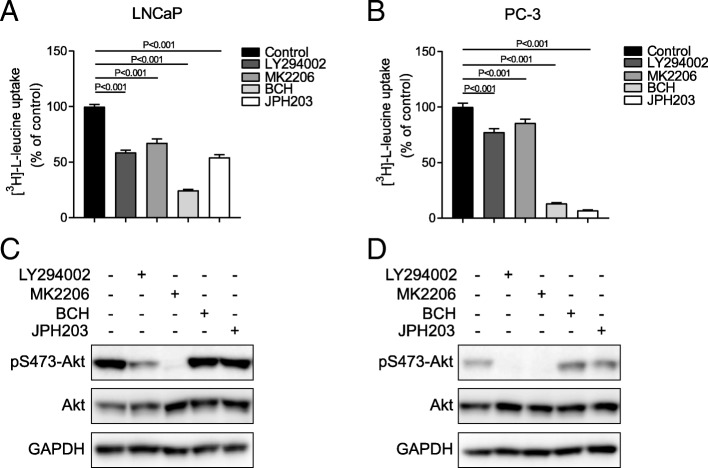
Leucine transport is regulated by PI3K/Akt signalling. **a** and **b**, leucine transport was examined in the presence of LY294002, MK2206, BCH and JPH203 in LNCaP (**a**) and PC-3 cells (**b**). One-way ANOVA was performed. Data are the mean ± SEM, *n* = 4. **c** and **d**, Akt activation was examined in the presence of LY294002, MK2206, BCH or JPH203 in LNCaP (**c**) and PC-3 cells (**d**) by western blot. GAPDH was used as the loading control. The densitometry of pAkt was normalised to Akt. All western blot images are representative of three independent experiments

To model the effects of growth factors on prostate cancer, we utilized the androgen-sensitive prostate adenocarcinoma cell line LNCaP, which contains one deleted allele and one mutated allele of PTEN [27], and the androgen-insensitive cell line PC-3, which has a homozygous deletion of PTEN [27, 28], leading to the hyperactivation of the PI3K/Akt pathway. Leucine uptake is predominantly mediated by LAT3 in LNCaP cells, while PC-3 cells rely on both LAT3 and LAT1 (SLC7A5) for leucine uptake [23]. To investigate how PI3K/Akt signalling affects LAT3-mediated leucine uptake in the presence of serum, we utilized a PI3K inhibitor LY294002, an Akt inhibitor MK2206, an L-type amino acid transporter inhibitor BCH and a LAT1 selective inhibitor JPH203 [29–32]. In LNCaP cells, leucine transport was inhibited to 59% of control in the presence of LY294002, 67% of control with MK2206 and 54% of control with JPH203, whereas BCH reduced leucine uptake to 24% of control (Fig. 1a). In PC-3 cells, the leucine transport was reduced to 81% of control by LY294002, and 94% of control by MK2206, which exhibited less inhibitory effect compared to BCH (12% of control) and JPH203 (6% of control; Fig. 1b), consistent with the dominant role of LAT1 in mediating leucine uptake in PC-3 cells [23]. Western blots showed that both LY294002 and MK2206 reduced Akt phosphorylation (pS473-Akt) in LNCaP and PC-3 cells after 30 min treatment. The LAT inhibitors, BCH and JPH203, had no effect on Akt phosphorylation (Fig. 1c, d). These results suggest that PI3K/Akt signalling pathway regulates leucine uptake, which is more likely through LAT3 rather than LAT1.

### LAT3 is required for PI3K/Akt regulated leucine uptake

**Fig. 2:**
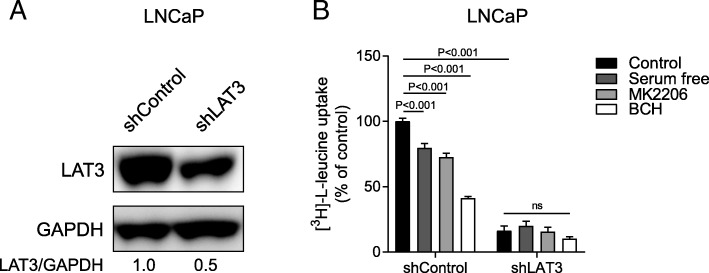
LAT3 is required for PI3K/Akt stimulated leucine uptake. **a** LAT3 expression levels were examined by western blot in shControl and shLAT3 of LNCaP cells. All western blot images are representative of three independent experiments. **b** leucine uptake was examined in RPMI supplemented with dialyzed FBS, serum-free media, MK2206 or BCH, in both shControl and shLAT3 of LNCaP cells. Two-way ANOVA was performed. Data are the mean ± SEM, *n* = 3

To determine if LAT3 is the target of PI3K/Akt signalling, we used shRNA to knock down LAT3 expression in LNCaP cells, which have higher LAT3 expression compared to PC-3 cells [23]. Western blots showed that LAT3 expression levels were decreased in LNCaP shLAT3 comparing to shControl (Fig. 2a). Leucine uptake assays were performed in both LNCaP shControl and shLAT3 cells. RPMI media supplemented with dialyzed FBS was used as control, and was compared to serum-free media, or addition of MK2206 or BCH. Dialyzed FBS contains normal level of growth factors but low levels of amino acids. In shControl cells, leucine uptake was reduced in serum-free media, MK2206 and BCH treatment groups (Fig. 2b). Under the same conditions, knockdown of LAT3 in LNCaP cells showed a significant reduction in leucine uptake equivalent to 16% of control in shControl cells (Fig. 2b), suggesting that LAT3 has been efficiently knocked down in LNCaP cells. Serum depletion and inhibition by MK2206 for shLAT3 cells showed no significant difference in leucine uptake compared to control (Fig. 2b), indicating that these signalling pathways activate leucine uptake in a LAT3-dependent manner (Fig. 2b).

### EGF stimulation regulates leucine uptake

**Fig. 3:**
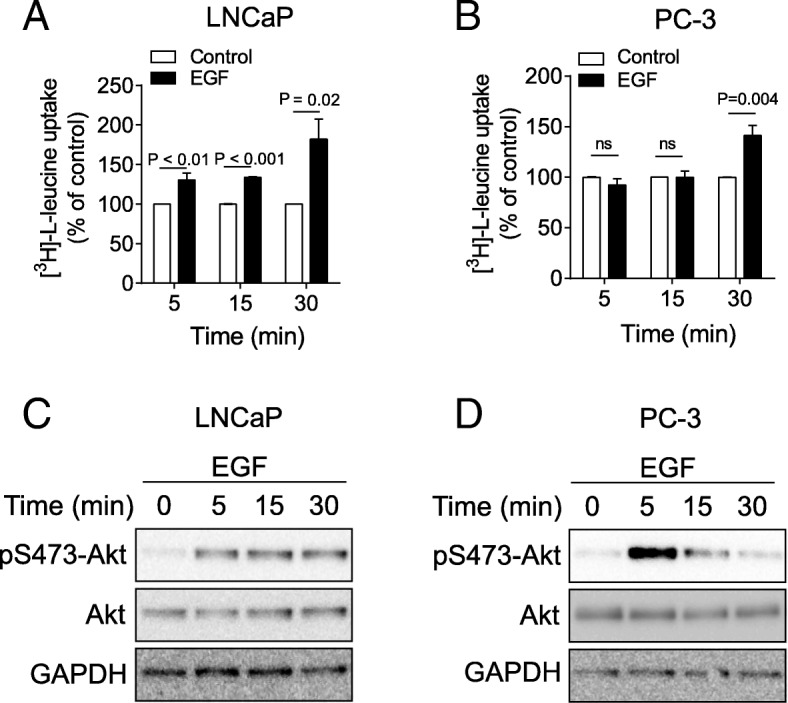
EGF stimulation increases leucine uptake. **a** and **b**, leucine uptake was examined 5, 15 or 30 min after EGF stimulation of LNCaP (**a**) or PC-3 cells (**b**). Two-tailed student’s t-test was performed. Data are the mean ± SEM, *n* = 4. **c** and **d**, the phosphorylation of Akt was examined 5, 15 or 30 min after EGF stimulation of LNCaP (**c**) or PC-3 cells (**d**). GAPDH was used as loading control. All western blot images are representative of three independent experiments

To specifically test whether EGF mediates activation of PI3K/Akt signalling pathway and leucine uptake, EGF treatment was carried out on LNCaP and PC-3 cells. EGF stimulation for 5, 15 or 30 min significantly increased leucine uptake in LNCaP cells by 30%, 33% and 79%, respectively (Fig. 3a). In PC-3 cells, there was no change in leucine uptake at either 5 or 15 min post EGF, however, there was a significant increase after 30 min (Fig. 3b). To determine whether this corresponded with downstream activation of signalling from the EGF receptor, we next examined the level of phosphorylated Akt (pS473-Akt) over the same time course. In LNCaP cells, Akt phosphorylation increased by 5 min, and remained at similar levels between 15 min and 30 min (Fig. 3c). PC-3 cells also rapidly increased Akt phosphorylation at 5 min, however, this phosphorylation was downregulated by 15 and 30 min post stimulation (Fig. 3d).

### Leucine transport is dependent on EGF-activated PI3K/Akt signalling

**Fig. 4:**
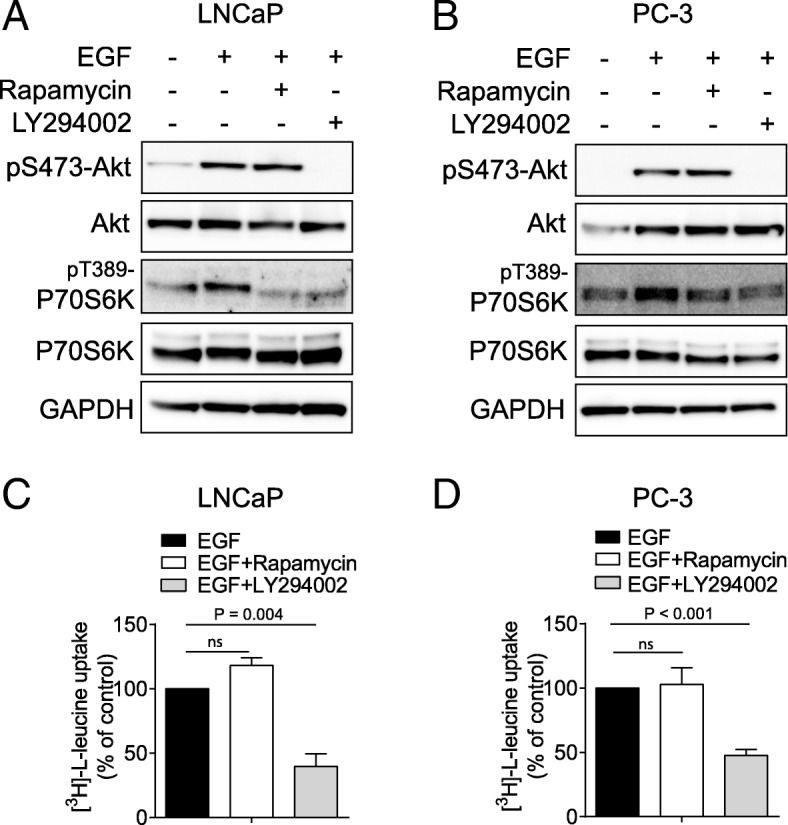
Leucine transport is dependent on EGF-stimulated PI3K/Akt signalling. **a** and **b**, Akt and P70S6K activation was examined in the presence of rapamycin and LY294002 in LNCaP (**a**) or PC-3 cells (**b**). GAPDH was used as loading control. All western blot images are representative of three independent experiments. **c** and **d**, leucine transport was examined in the presence of rapamycin or LY294002 in LNCaP (**c**) and PC-3 cells (**d**). Two-tailed student’s t-test was performed. Data are the mean ± SEM, *n* = 3

EGF stimulates the PI3K/Akt signalling pathway, subsequently activating mTORC1 signalling and driving protein synthesis. To determine which kinase in the PI3K/Akt/mTORC1 pathway axis was involved, we tested a PI3K inhibitor, LY294002, and an mTORC1 inhibitor, rapamycin, in combination with EGF treatment. As expected, 30 min pre-incubation with LY294002 blocked the activation of both Akt and downstream P70S6K (Fig. 4a, b) signalling, while rapamycin treatment decreased phosphorylation of P70S6K (Fig. 4a, b), but had no effect on upstream Akt phosphorylation. In addition, while LY294002 treatment significantly reduced leucine transport in both LNCaP and PC-3 cells (Fig. 4c, d), rapamycin showed no effect on leucine uptake in either cell line (Fig. 4c, d). These data suggest that the PI3K/Akt signalling pathway can regulate leucine transport, and this occurs upstream of mTORC1 signalling.

### EGF stimulation promotes co-localisation of LAT3 with pAkt

**Fig. 5:**
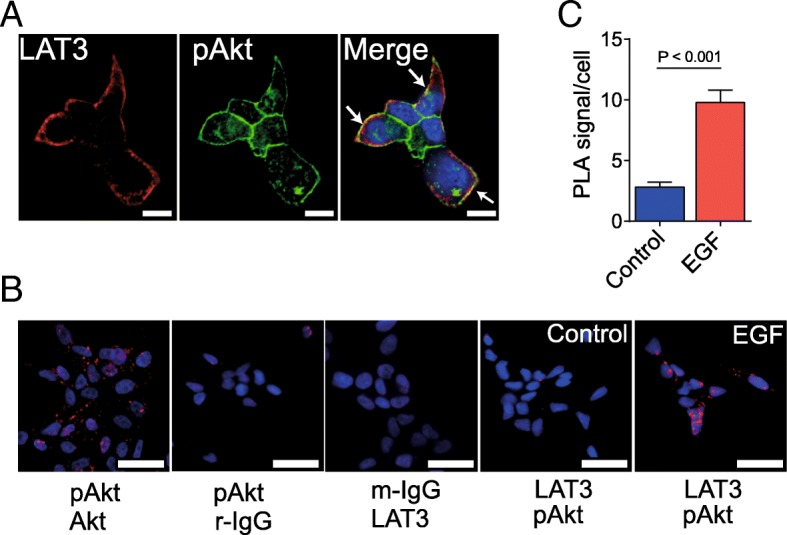
Co-localization of LAT3 and pAkt in LNCaP cells. **a** co-localization of LAT3 and pAkt was examined in LNCaP cells in the presence of EGF. Scale bar = 10 μm. **b** PLA signals were examined in the pair of mouse pAkt and rabbit Akt as positive control, mouse pAkt and rabbit-IgG (r-IgG), mouse-IgG (m-IgG) and rabbit LAT3 as negative control, rabbit LAT3 and mouse pAkt (Control), and LAT3 and pAkt with EGF (EGF). Scale bar = 50 μm. **c** quantification of PLA signals in the absence or presence of EGF in LNCaP cells was measured using Volocity. One-way ANOVA was performed. Data are the mean ± SEM, *n* = 4

To determine the relationship between PI3K/Akt signalling and LAT3, we examined the co-localisation of phosphorylated Akt and LAT3 using confocal microscopy in LNCaP, as LAT3 is highly expressed in LNCaP cells but not in PC-3 cells. LAT3 was strongly expressed at the plasma membrane of cells that were in contact with the extracellular environment, but showed lower expression in areas of cell-cell contact (Fig. 5a). By contrast, pAkt was detected in the majority of the plasma membrane of LNCaP cells (Fig. 5a). The merged image showed a number of areas of co-localisation of LAT3 and pAkt (Fig. 5a, arrows), as well as distinct areas of single staining of each protein/phosphoprotein.

To confirm these confocal microscopy data, we next performed a proximity ligation assay (PLA) to determine whether pAkt and LAT3 were closely associated in LNCaP cells. To demonstrate the value of this technique, mouse pAkt and rabbit Akt antibodies bound to different antigens on the same target, resulted in a high co-localisation signal (Fig. 5b). Next, we used rabbit IgG with mouse pAkt antibody or rabbit LAT3 antibody with mouse IgG as negative controls to establish background PLA signals (Fig. 5b). LNCaP cells demonstrate a low level basal amount of PLA signal with LAT3 and pAkt, however after EGF treatment the PLA signals of LAT3 and pAkt were dramatically enhanced (Fig. 5b, c), indicating that EGF enhanced the co-localisation of phosphorylated Akt and LAT3. Given the fact that PLA resolution is within 40 nm [33, 34] pAkt and LAT3 were in close proximity and they may form a multi-protein complex in response to the extracellular signals as well as to increase amino acid uptake required for cell division.

### EGF stimulation enhanced surface expression of LAT3

**Fig. 6:**
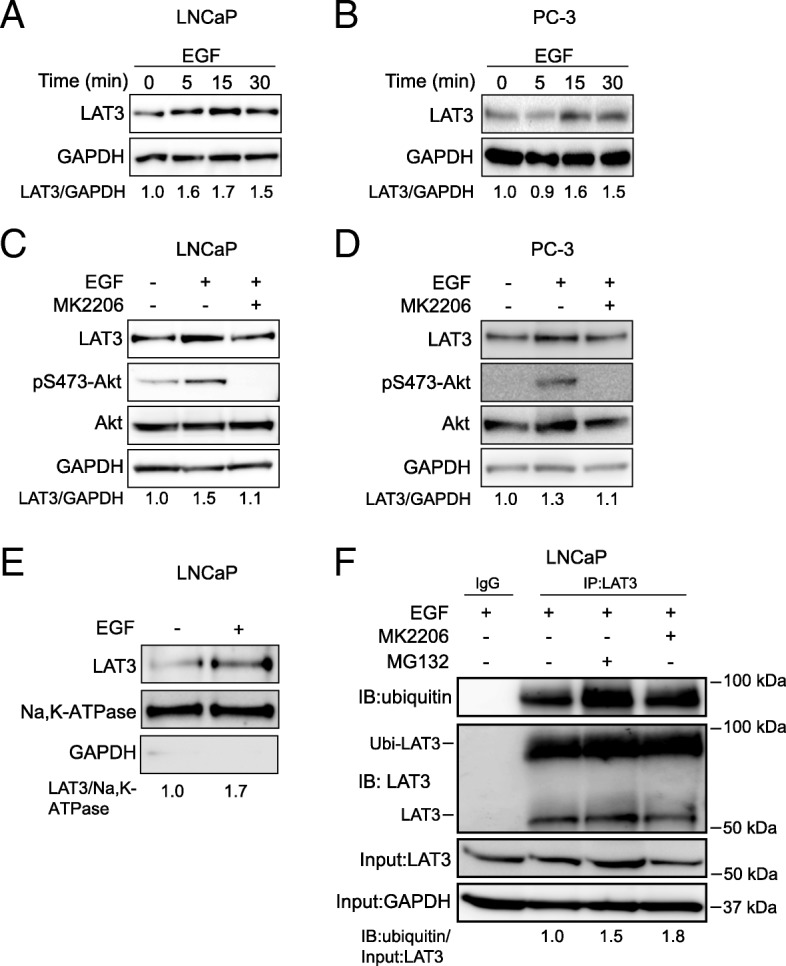
EGF treatment increases LAT3 expression on the plasma membrane. **a** and **b**, LAT3 expression levels were examined 5, 15 or 30 min after EGF stimulation of LNCaP (**a**) or PC-3 cells (**b**). **c** and **d**, LAT3 and Akt expression levels were examined in the presence or absence of MK2206 in combination with EGF in LNCaP (**c**) or PC-3 cells (**d**). GAPDH was used as loading control. **e** LAT3 protein levels were examined in the absence or presence of EGF in LNCaP cells by cell surface protein isolation; Na, K-ATPase was used as loading control. GAPDH was used to confirm cell surface fraction purity. **f** Ubiquitin and LAT3 were examined after immunoprecipitation with isotype IgG or anti-LAT3 IgG in the presence or absence of MK2206 or MG132 with EGF stimulation in LNCaP cells. Input of cell lysates pre-immunoprecipitation was examined using LAT3 and GAPDH antibodies. Ratio of IB: ubiquitin expression level is shown relative to Input: LAT3. All western blot images are representative of three independent experiments

To determine the mechanism of PI3K/Akt regulated leucine transport via LAT3, we examined LAT3 expression by western blots in the presence of EGF. LAT3 protein levels were elevated within 5 min of EGF treatment and maintained for 30 min in LNCaP cells (Fig. 6a). In PC-3 cells, LAT3 protein levels increased at 15 min and maintained this increase for 30 min (Fig. 6b), indicating that EGF-induced leucine uptake may result from upregulated LAT3 surface expression. This late elevation of LAT3 in PC-3 cells might explain why changes in leucine uptake are delayed, although this may also be due to physical differences in the analysis of adherent (western blot) and suspension (uptake assay) cells (Fig. 3b). As LAT1 is the major transporter in PC-3 cells [23], we examined LAT1 expression after EGF treatment. LAT1 protein levels did not change after 30 min EGF treatment, suggesting that LAT1 is not involved in the EGF-stimulated upregulation of leucine transport (Additional file 1: Figure S1A). To confirm this, we next examined EGF stimulation of leucine transport in the presence of the LAT1 inhibitor JPH203 (Additional file 1: Figure S1B). Despite blocking LAT1-mediated leucine transport, JPH203 had no effect on the EGF-stimulated leucine uptake, confirming that LAT1 does not play a role in EGF stimulation of PC-3 cells. Importantly, the Akt inhibitor MK2206 suppressed Akt phosphorylation as well as LAT3 expression even in the presence of EGF in LNCaP and PC-3 cells (Fig. 6c, d), suggesting EGF-activated Akt is required for increased LAT3 expression.

Since LAT3 needs to be stabilized at the plasma membrane to fulfil its transport function, the expression level at cell surface is critical in response to EGF stimulation. Cell surface proteins were isolated and LAT3 was examined by western blot in the presence or absence of EGF. After 30 min treatment with EGF, LAT3 expression was increased 1.7-fold at the plasma membrane compared to control (Fig. 6e and Additional file 1: Figure S1C), suggesting that LAT3 levels were increased at the cell surface. To determine whether EGF treatment affects either LAT3 synthesis or LAT3 degradation, we next treated EGF-stimulated cells with MK2206 or a proteasome inhibitor MG132, which reduces the ubiquitin-mediated degradation of protein in the proteasome. After 30 min MG132 and MK2206 treatment, ubiquitinated LAT3 (~ 90 kDa) was increased 1.5-fold and 1.8-fold in the anti-LAT3 immunoprecipitates respectively (Fig. 6f). In addition, we showed that ubiquitinated LAT3 (~ 90 kDa) is decreased after EGF treatment in both LNCaP and PC-3 cells (Additional file 1: Figure S1D, E). After MG132 treatment, unmodified LAT3 protein levels (~ 55 kDa) are increased compared to control (Additional file 1: Figure S1F). These results suggest that inhibition of EGF-activated PI3K/Akt signalling may induce ubiquitin-mediated degradation of LAT3.

## Discussion

Rapidly proliferating cancer cells require sustained growth factor stimulation and more nutrient supply for protein synthesis and cell mass accumulation [2]. Growth factors, such as EGF, activate multiple downstream signalling pathways and have an important role in cancer progression, including proliferation, invasion and migration [35]. Its downstream PI3K/Akt signalling, and amino acids are required for mTORC1 to regulate protein synthesis. In this study, we firstly reported that EGF-activated PI3K/Akt signalling pathway regulates leucine uptake through the amino acid transporter LAT3 in prostate cancer.

Cells have many ways of uptaking nutrients that are available in the extracellular matrix, such as transporter-based nutrient uptake, receptor-mediated endocytosis, macropinocytosis, as well as entosis [36]. The transport of majority extracellular amino acids relies on their membrane-bound transporters due to their hydrophilicity, and the expression of transporters is highly upregulated in many cancer types, such as Alanine-Serine-Cysteine Transporter 2 (ASCT2/SLC1A5) in triple negative breast cancer, prostate cancer and melanoma [37–39], LAT1 (SLC7A5) in endometrial cancer [40], ATB^0,+^ (SLC6A14) in colorectal cancer [41], xCT (SLC7A11) in glioma [42]. Growth factor signalling pathways play an instructive role in regulating nutrient transporters via PI3K/Akt, which induces the phosphorylation and translocation of glucose transporter 1 (GLUT1) to cell surface to increase glucose uptake upon growth factor stimulation [43, 44]. PI3K/Akt signalling also affects transcription factor MYC, which induces the expression of glutamine transporters ASCT2 and SNAT5 and promotes glutaminolysis to provide glutamine carbon for the TCA cycle in response to growth factor [45, 46]. A recent study has showed that active MYC could preferentially upregulate LAT3 among many other SLC transporters in neuroblastoma cells, and that inhibition of LAT3 would in turn downregulate MYC mRNA levels [47]. In this study, we have observed that total and surface LAT3 protein levels are increased within 5–30 min after EGF stimulation. When LAT3 is knocked down in LNCaP cells, leucine transport becomes less sensitive to the stimulation or inhibition of the signalling pathway. Importantly, ubiquitinated LAT3 levels (~ 90 kDa) are increased after inhibition PI3K/Akt signalling, suggesting that EGF-activated PI3K/Akt signalling may affect the protein stabilisation or localisation of LAT3 to maintain its activity, therefore regulating leucine uptake. Activated Akt has been shown to be able to maintain the surface expression of other transporters [48], which is consistent with what we observed in this study.

Akt, as a major effector in PI3K/Akt signalling axis, has an important role in activating mTORC1 signalling. Recent studies have shown that inhibition of mTORC1 signalling increases ubiquitin ligase Nedd4-2 expression, therefore upregulating ubiquitination of SNAT2 and LAT1 transporters in primary human trophoblast cells [25]. However, our data clearly show that LAT3-mediated leucine transport does not rely on downstream mTORC1 signalling, but instead is regulated by Akt or other upstream molecules. Akt contains a Pleckstrin homology (PH) domain which binds to phosphoinositides on the plasma membrane [49], thereby allowing it to phosphorylate several multi-transmembrane proteins, such as G protein coupled receptor EDG-1 [50], and Na^+^/H^+^ exchanger SLC9A1 (NHE1) [51]. Our data showed that phosphorylated Akt co-located with LAT3 at the plasma membrane of LNCaP cells. Although mass spectrometry data have shown that LAT3 possesses multiple phosphorylation sites at Y251, S262 and S267, and one ubiquitination site at K264 within its transmembrane domains [52–54], we have no direct evidence to show that Akt binds and phosphorylates LAT3 directly. It is possible that LAT3 and Akt are part of a larger membrane-localised protein complex formed after EGF stimulation. Subsequent Akt activation in that complex may induce another kinase to phosphorylate LAT3 at residues S262 or S267. This may then inhibit K264 ubiquitination, thus permitting LAT3 stabilisation at the plasma membrane.

Our study emphasizes a central role of growth factor activated PI3K/Akt signalling in response to environment changes. With growth factor stimulation, PI3K/Akt signalling increased stabilisation of the LAT3 transporter and its localisation on the plasma membrane, thereby enhancing leucine transport. Elevated intracellular levels of leucine are then available for mTORC1 signalling activated by PI3K/Akt signalling. At low level growth factor-stimulation, our data suggest that more LAT3 will be degraded and less proteins recycled to the plasma membrane, resulting in decreased leucine transport and protein synthesis. This would be an economic strategy for cells, while still allowing rapid upregulation of leucine transport upon growth factor stimulation.

## Conclusion

This is the first study to show that growth factor-activated PI3K/Akt signalling pathway regulates leucine transport through LAT3 in prostate cancer cell lines. These data support a direct link between growth factor and amino acid uptake, providing a mechanism by which the cells rapidly coordinate amino acid uptake for cell growth. Previous studies have shown that LAT3 protein expression is increased in the primary and recurrent prostate cancer patient samples and regulates cancer cell growth [23, 24]. In addition, LAT3 is expressed in liver, pancreas and muscle cells [22]. Leucine is critical for insulin secretion in pancreatic β cells [55]. LAT3 is also important for podocyte development and function in kidney [56]. Therefore, our study on the regulatory mechanisms of LAT3 is important for understanding the metabolism of leucine across many systems, and potentially for developing novel cancer therapies targeting the LAT3 transporter.

## Additional file

Additional file 1: **Figure S1.** A, LAT1 expression after EGF treatment in PC-3 cells. GAPDH was used as loading control. B, leucine uptake in PC-3 cells after JPH203 treatment in the absence or presence of EGF for 30 min. Two tailed Student’s t-test was performed. Data are the mean ± SEM, *n* = 4. C, GAPDH is examined in cell surface fraction in LNCaP cells. Cell lysates of LNCaP cells are used as positive control. D and E, Ubiquitin and LAT3 were examined after immunoprecipitation with anti-LAT3 in the presence or absence of EGF in LNCaP (D) and PC-3 (E) cells. Ratio of IB: ubiquitin expression level is shown relative to Input: LAT3. F, LAT3 expression levels were examined in the absence or presence of MG132 in EGF treated LNCaP or PC-3 cells. GAPDH was used as loading control. Protein expression levels were normalised to loading control. (PDF 1131 kb)

## Data Availability

All data generated or analyzed during this study are included in this published article.

## Abbreviations

BCAA: Branched chain amino acid
EGF: Epidermal growth factor
EGFR: Epidermal growth factor receptor
GAP: GTPase activating protein
LAT3: L-type amino acid transporter 3
MAPK: Mitogen-activated protein kinase
mTORC1: Mechanistic target of rapamycin complex 1
PBS: Phosphate buffered saline
PI3K: Phosphoinositide 3 kinase
PIP2: Phosphatidylinositol (3,4)-bisphosphate
PIP3: Phosphatidylinositol (3,4,5)-trisphosphate
PLA: Proximity ligation assay
PTEN: Phosphatase and tensin homolog
SDS-PAGE: Sodium dodecyl sulfate polyacrylamide gel electrophoresis
SLC43A1: BCH: 2-amino-bicyclo [2,2,1] hepta-2-carboxylic acid
TSC1/2: Tuberous sclerosis complex 1/2
