# Supplementary material for: EGF-activated PI3K/Akt signalling coordinates leucine uptake by regulating LAT3 expression in prostate cancer
Source: Cell Commun Signal. 2019 Jul 25;17:83. doi: 10.1186/s12964-019-0400-0 (PMC6659227; doi:10.1186/s12964-019-0400-0)
Supplement: Supplementary file 1 — Figure S1. A, LAT1 expression after EGF treatment in PC-3 cells. GAPDH was used as loading control. B, leucine uptake in PC-3 cells after JPH203 treatment in the absence or presence of EGF for 30 min. Two tailed Student’s t-test was performed. Data are the mean ± SEM, n = 4. C, GAPDH is examined in cell surface fraction in LNCaP cells. Cell lysates of LNCaP cells are used as positive control. D and E, Ubiquitin and LAT3 were examined after immunoprecipitation with anti-LAT3 in the presence or absence of EGF in LNCaP (D) and PC-3 (E) cells. Ratio of IB: ubiquitin expression level is shown relative to Input: LAT3. F, LAT3 expression levels were examined in the absence or presence of MG132 in EGF treated LNCaP or PC-3 cells. GAPDH was used as loading control. Protein expression levels were normalised to loading control. (PDF 1131 kb) [file 12964_2019_400_MOESM1_ESM.pdf]

Supplementary Figure 1

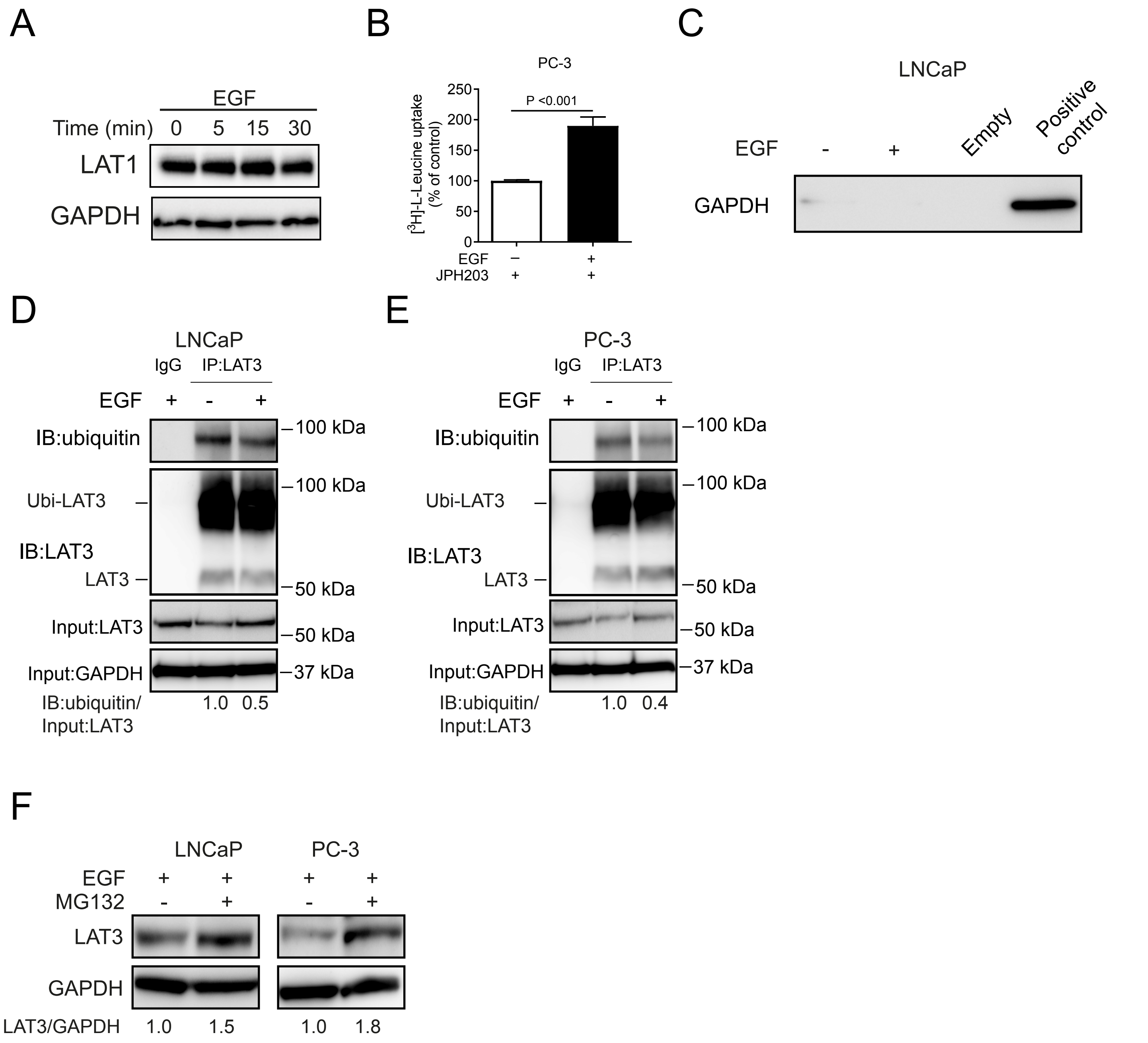

Supplementary Figure 1: **A**, LAT1 expression after EGF treatment in PC-3 cells. GAPDH was used as loading control. **B**, leucine uptake in PC-3 cells after JPH203 treatment in the absence or presence of EGF for 30 min. Two tailed Student's t-test was performed. Data are the mean ± SEM, n=4. **C**, GAPDH is examined in cell surface fraction in LNCaP cells. Cell lysates of LNCaP cells are used as positive control. **D** and **E**, Ubiquitin and LAT3 were examined after immunoprecipitation with anti-LAT3 in the presence or absence of EGF in LNCaP (**D**) and PC-3 (**E**) cells. Ratio of ubiquitin expression level is relative to Input: LAT3. **F**, LAT3 expression levels were examined in the absence or presence of MG132 in EGF treated LNCaP or PC-3 cells. GAPDH was used as loading control. Protein expression levels were normalised to loading control.
